# Near-Complete Avipoxvirus Genome Assembled from Skin Lesions of Dead Eurasian Crane (*Grus grus*)

**DOI:** 10.3390/ani15010060

**Published:** 2024-12-30

**Authors:** Eszter Kaszab, Endre Sós, Krisztina Bali, Viktória Sós-Koroknai, Edina Perge, Krisztina Ursu, Szilvia Marton, Márton Hoitsy, Gábor Kemenesi, Krisztián Bányai

**Affiliations:** 1 HUN-REN Veterinary Medical Research Institute, Tábornok u. 2., H-1143 Budapest, Hungary; eszter.kaszab@gmail.com (E.K.); krisz.bali@gmail.com (K.B.); marton.szilvia@vmri.hun-ren.hu (S.M.); 2 Department of Microbiology and Infectious Diseases, University of Veterinary Medicine Budapest, H-1078 Budapest, Hungary; 3 One Health Institute, University of Debrecen, H-4032 Debrecen, Hungary; 4 National Laboratory for Infectious Animal Diseases, Antimicrobial Resistance, Veterinary Public Health and Food Chain Safety, H-1143 Budapest, Hungary; 5 Budapest Zoo and Botanical Garden, Állatkerti krt. 6-12, H-1146 Budapest, Hungary; drsos.endre@zoobudapest.com (E.S.); koroknai.viktoria@zoobudapest.com (V.S.-K.); hoitsy.marton@zoobudapest.com (M.H.); 6 Department of Exotic Animal and Wildlife Medicine and Clinic, University of Veterinary Medicine, H-1078 Budapest, Hungary; 7 Matrix Histopathology Service, Váci út 174, H-1085 Budapest, Hungary; eperge@gmail.com; 8 Veterinary Diagnostic Directorate, National Food Chain Safety Office, H-1024 Budapest, Hungary; ursu.krisztina@gmail.com; 9 Institute of Biology, Faculty of Sciences, University of Pécs, H-7624 Pécs, Hungary; kemenesi.gabor@pte.hu; 10 National Laboratory of Virology, Szentágothai Research Centre, University of Pécs, H-7624 Pécs, Hungary; 11 Department of Pharmacology and Toxicology, University of Veterinary Medicine, H-1078 Budapest, Hungary; 12 Department of Laboratory Medicine, Medical School, University of Pécs, H-7624 Pécs, Hungary; 13 Molecular Medicine Research Group, Szentágothai Research Centre, University of Pécs, H-7624 Pécs, Hungary

**Keywords:** avipoxvirus, genome sequencing, phylogenetic analysis

## Abstract

Avian pox is a globally spread viral disease affecting a wide spectrum of wild and domesticated bird species. The disease is caused by a diverse group of large DNA viruses, namely, avipoxviruses (genus *Avipoxvirus*, family *Poxviridae*). In this study, gross pathological examination and histopathological examination of skin lesions and several organs suggested acute poxvirus infection of a Eurasian crane (*Grus grus*, Linnaeus, 1758). Avipoxvirus infection was confirmed by testing wart-like lesions via gene-specific PCR assay and sequencing the obtained amplicon. Phylogenetic analysis of the gene encoding the DNA polymerase revealed that the crane poxvirus clustered in clade A, subclade A3. A large fragment of the poxvirus genome (306,477 bp in length) was assembled from the DNA of a skin specimen. Our study reaffirms previous findings that even complex virus genomes can be determined from a metagenomic assemblage generated directly from avian tissue samples without prior virus isolation, a promising approach for the epidemiologic surveillance of avipoxvirus infections in wild birds and domestic poultry.

## Simple Summary

Avipoxviruses are considered emerging threats to the poultry industry and wildlife. In wild birds, avian pox may cause mass mortality, affecting ongoing species conservation efforts. Monitoring the circulating avipoxvirus strains help us to better understand the ecology, epizootiology, and evolution of poxvirus infections in wild birds. In this study, we constructed a near-complete poxvirus genome from the skin lesions of a dead Eurasian crane without the need for prior virus isolation. Our findings reinforce the hypothesis that the metagenomic assemblage from wart-like lesions could be an ideal source of sequence data for avipoxvirus genome assembly.

## 1. Introduction

Avian pox is a viral disease affecting over 370 bird species across 76 families and 23 orders of birds worldwide [1,2,3]. In addition to the economic losses caused by avian pox in domestic poultry, the virus is also responsible for morbidity and mortality in wildlife, challenging species conservation efforts. Two common forms of the disease can develop in infected birds. One manifestation is the cutaneous or dry form, characterized by wart-like growths, primarily on non-feathered body areas (such as legs, feet, beak, and eyes) [1,3,4]. The other form is the diphtheritic or wet form, characterized by lesions and yellowish crusts on the mucous membranes of the lungs, trachea, esophagus, and mouth. This form is usually responsible for a higher mortality rate [1,2,3,4]. Avipoxviruses have two main routes of transmission. The arthropod-mediated transmission through the bites of midges, mites, and mosquitoes is predominantly responsible for the cutaneous form of the disease. Avipoxviruses can be also transmitted via the inhalation or ingestion of dust and aerosols or through direct contact with contaminated objects, such as feeders and perches [3,5,6].

Poxviruses of birds were identified as causative agents of pox lesions more than 150 years ago [4]. Poxviruses are amongst the largest animal viruses; they are characterized by brick-shaped virion of 330 nm and a linear, double-stranded DNA genome of 250–400 kb in length, which encodes 250–300 proteins [3]. Avian poxviruses belong to the genus *Avipoxvirus*, subfamily *Chordopoxvirinae*, family *Poxviridae*. At present, seven species are listed within the genus according to the latest report of the International Committee on the Taxonomy of Viruses [7]. Phylogenetic analysis classifies avipoxviruses into five genetic clades (designated A to E) and multiple sub-clades within the major clades (such as clade A and clade B) [5,8].

The distribution of clades and sub-clades in different hosts and geographic areas was first summarized in a multicenter study by Gyuranecz and co-workers [8]. The analysis of >100 viral sequences originating from avipoxvirus isolates or pathological samples has revealed that subclade A1 and A2 avian poxviruses can be isolated mainly from Galliformes and Columbiformes, respectively. Subclade A3 strains have been detected in numerous sea birds (e.g., petrels, cormorants, and penguins) in the Pacific and Atlantic Ocean areas, but it has also occurred in an owl species, whereas subclades A4 and A5 have been found to infect falcons in Eurasia and Anseriformes in the Americas, respectively. In this study, which has uncovered new contexts with respect to phylogenetics and the phylogeography of avian poxvirues, two novel subclades, A6 and A7, have been identified: subclade A6 has been detected in some North American Columbiformes; whereas subclade A7 has been identified in Accipitriformes. Additionally, both novel subclades have been identified in Anseriformes. The avipoxvirus subclade B1 has been detected in passeriform birds worldwide and also in some other non-passerine species (e.g., cranes, penguins). Subclade B2 viruses have been detected in Europe and North America from birds belonging to different taxa (such as bustards, pigeons, and songbirds), whereas subclade B3 strains have been described from songbirds and waders from North America. At present, clade C avian poxviruses are known to infect psittacine birds, whereas clade D and E avian poxviruses appear to infect selected species within Galliformes in Europe, Africa, and South America [9,10,11].

The molecular identification and characterization of avian poxviruses has been based on PCR amplification and DNA sequencing of partial genomic regions, mainly the 4b protein and the DNA polymerase coding region [8]. Indeed, the majority of GenBank entries are limited to these short genomic regions, whose length is less than one percent of the entire avian poxvirus genome. Recently, whole-genome sequencing has become a widely available method due to the spread of low-cost benchtop sequencers and the outreach of sequencing services. Shotgun metagenomics of biological samples containing viruses requires mid-to-high-throughput sequencers, whose outputs could, in theory, be sufficient to assemble virus genomes of various sizes. The potential of this approach to generate large fragments or (near-)complete virus genomes from clinical/pathological specimens is very attractive and accelerates the diagnostics of new viral diseases and supports the epidemiological monitoring of emerging viruses. The field of avian virology has benefited from the advantages of next-generation sequencing methods in recent years, as exemplified by studies describing the rapid generation of new genome sequences from various biological samples, including fecal specimens and tissue samples [12,13].

In this study, the pathology and virology findings of a lethal avian pox case in a dead specimen of Eurasian crane (*Grus grus*) is described. By using shotgun metagenomics, we assembled the near full-length viral genome directly from a pathological sample without prior virus isolation.

## 2. Materials and Methods

### 2.1. Case Description

The bird was transported to the Wildlife Rescue Centre of the Budapest Zoo and Botanical Garden from the Hortobágy (located in eastern Hungary) in late 2019. Hortobágy is a large natural area where migratory birds visit grasslands, fishponds, and wetlands. The Eurasian crane, like many other migratory birds, uses the Hortobágy as a mainland roosting place during its European migration. After the bird died in November, an autopsy was carried out and samples were collected for histological and virological examination.

**Figure 1:**
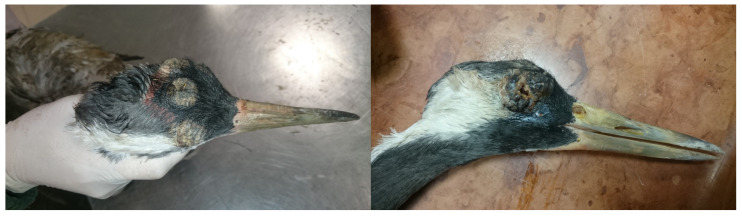
Severe wart-like lesions on the head of a Eurasian crane (*Grus grus*).

The crane was an adult specimen and measured 3.5 kg. The skin and the feathers of the bird were normal, except on the head. Wart-like lesions were seen on the top of the head and on the eye lids (Figure 1). In the opened body cavity, the wall of the air sacs was clear, and the lung was hyperemic. The kidneys were pale and enlarged, the liver was enlarged and the edges were rounded, and the gall bladder was filled with bile. The heart was pale. The spleen was enlarged and showed signs of septicemia. The lumen of the intestine was empty.

### 2.2. Histology

For histopathological examination, samples from organs (kidney, liver, heart muscle, intestinal wall, lung, spleen, and skin) were placed into 10% buffered formalin for fixation and were kept in the fixative at room temperature for 24 h. Then, the samples were processed with an automated tissue sample preparation system (HistoCore PEGASUS Plus Tissue Processor; Leica Biosystems, Nussloch, Germany; https://www.leicabiosystems.com/histology-equipment/tissue-processors/histocore-pegasus-plus-tissue-processor/, accessed on 2 December 2024). Slices, which were 3–4 μm thick, were prepared from the paraffin-embedded tissue blocks and stained with haematoxylin–eosin. The stained slides were examined using a Euromex BioBlue BB.4260 light microscope and scanned with Panoramic MIDI II (3DHistech); representative pictures were taken by a CaseViewer (3DHistech).

### 2.3. Virus PCR and Amplicon Sequencing

Tissue samples were homogenized in PBS by adding stainless steel beads and running for 15 min at 50 Hz on TissueLyzer LT (Qiagen, Hilden, Germany). Then, the homogenized samples were centrifuged at 10,000× *g* for 5 min. Total DNA was purified directly from tissue lysates using a ZiXpress-32^®^ Automated Nucleic Acid Purification Instrument and ZiXpress-32^®^ Viral Nucleic Acid Extraction Kit (Zinexts Life Science Corp., New Taipei City, Taiwan).

The partial DNA polymerase gene was amplified by PCR using the following primer pairs: PoPr1 (5′-CGCCGCATCATCTACTTATC-3′) combined with PoPr2 (5′-CCACACAGCGCCATTCATTA-3′) and PPolF (5′-GGCYAGTACKCTTATYAAAGG-3′) in combination with PPolR (5′-CGTCTCTACGTGTTTCGCT-3′) [8]. Each PCR was conducted in a reaction volume of 25 µL containing 1 µL of the extracted the extracted nucleic acid, 200 nM of primers, 200 μM of dNTP mix, 1× DreamTaq Green buffer, and 0.625 U of DreamTaq DNA Polymerase (Thermo Scientific, Waltham, MA, USA). The PCRs contained the step of initial denaturation at 95 °C for 3 min, 40 cycles of denaturation at 95 °C for 30 s, primer annealing at 53 °C (for primer pair 1), and 50 °C (for primer pair 2) for 30 s and extension at 72 °C for 1 min, followed by a final extension step at 72 °C for 10 min.

The PCR products were purified from 1% agarose gel with Geneaid Gel/PCR Fragments Extraction Kit (Geneaid, Biotech, Taipei, Taiwan) and were directly sequenced with the BigDye Terminator v3.1 Cycle Sequencing Kit (Thermo Scientific, Waltham, MA, USA). Dye-labeled products were ethanol-precipitated, dried, and sent to a sequencing service based in Hungary for chromatographic analysis.

### 2.4. Whole Genome Sequencing

Next-generation sequencing was carried out using DNA extracted from the cutaneous warts. The Illumina^®^ Nextera XT DNA Library Preparation Kit (Illumina, San Diego, CA, USA) and the Nextera XT Index Kit v2 Set A (Illumina, San Diego, CA, USA) were used to prepare Illumina specific libraries [14,15]. In brief, DNA samples were diluted to 0.2 ng/μL in nuclease-free water (Promega, Madison, WI, USA) in a final volume of 2.5 μL. Reaction components were used at a reduced volume. For the tagmentation reaction, five microliters of Tagment DNA buffer with 2.5 μL of Amplicon Tagment Mix were used. During tagmentation, the samples were incubated at 55 °C for 6 min using the GeneAmp PCR System 9700 (Applied Biosystems/Thermo Fisher Scientific, Foster City, CA, USA). The samples were then allowed to cool to 10 °C before the addition of 2.5 μL of the Neutralize Tagment buffer. Neutralization was performed for 5 min at room temperature. A total of 7.5 μL of the Nextera PCR Master Mix and 2.5 μL each of the i5 and i7 index primers were added to the tagmented DNA samples. The index primers were incorporated into library DNA via 12 PCR cycles (each cycle consisted of the following steps: 95 °C for 10 s, 55 °C for 30 s, followed by 72 °C for 30 s). Following the PCR cycles, the samples were held at 72 °C for 5 min and then at 10 °C. Libraries were purified using the Gel/PCR DNA Fragments Extraction Kit (Geneaid Biotech Ltd., Taipei, Taiwan). The concentration of the purified libraries was measured; then, the libraries were pooled and denatured. The denatured library pool at a final concentration of 1.5 pM was loaded onto a NextSeq 500/550 Mid Output flow cell and sequenced using an Illumina^®^ NextSeq 500 sequencer (Illumina, San Diego, CA, USA).

### 2.5. Data Analysis

The viral DNA polymerase gene was analyzed using Bioedit v7.2.5 and the online BLAST tool to identify homologous strains [16,17]. The nucleotide sequences of DNA polymerase gene (partial and complete CDS, n = 198) related to the genus *Avipoxvirus* were downloaded from GenBank (https://www.ncbi.nlm.nih.gov/genbank/, accessed on 2 February 2024). Sequence alignment was carried out using MAFFT algorithm in Geneious Prime^®^ 2021.2.2 (https://www.geneious.com, accessed on 2 February 2024; Biomatters Ltd., Auckland, New Zealand). For phylogenetic analysis, genomes were trimmed manually, and a maximum likelihood phylogenetic tree was constructed using the GTR + G + I model with 1000 bootstrap replicates of the PhyML software (version 3.0) [18].

For whole-genome analysis, a quality check of the 150 bp single-end reads was performed using FastQC v.0.11.9 (https://www.bioinformatics.babraham.ac.uk/projects/fastqc/, accessed on 2 February 2024), and low-quality sequences and adaptors were removed using Cutadapt v.3.4 and fastp [19,20]. Then, the reads were corrected using Bloocoo [21]. Default parameters were used unless otherwise specified. The quality-filtered reads were assembled de novo using SPAdes v.3.15.3 [22], with error correction turned off. In parallel, mapping of raw reads was performed against reference genome of the Flamingopox virus FGPVKD09 (GenBank, MF678796.1) using Geneious Prime 2022.2.2 (https://www.geneious.com, accessed on 2 February 2024; Biomatters Ltd., Auckland, New Zealand). Contigs were manually edited by using Geneious Prime. The obtained complete genomes were annotated with Geneious Prime 2022.2.2. Additional open reading frames (ORFs) were predicted with the ORF Finder tool (https://www.ncbi.nlm.nih.gov/orffinder/, accessed on 2 February 2024).

The viral metagenomic sequence reads used to determine the genome of Avipoxvirus common crane-1 were deposited under the Bioproject ID PRJNA1074950 (other repository IDs were as follows: Biosample, SAMN39897821; SRA, SRR27940307; Genbank, PP341421).

## 3. Results

### 3.1. Histopathology and Laboratory Diagnosis

Specimens from various organs were prepared for histopathological examination. In the interstitial layer of the kidney, mixed inflammatory cell infiltration and necrosis were observed. Lymphocytes, plasma cells, and heterophilic granulocytes were involved in inflammatory infiltration. Large foci of basophilic particles, cyst-like nodes, were also found scattered in the interstitium and the ducts, and some basophilic particles could be found within macrophage cells. A conglomerate of similar particles without a capsule was also observed. In the heart preparation, multiple focal and mixed inflammatory cell infiltrations were seen in the myocardium, and necrosis was found in some inflammatory foci. The liver had a large number of basophilic foci similar to those described in the kidney, and an almost diffuse, mixed inflammatory cell infiltration—containing heterophils, lymphocytes, plasma cells, and macrophages—was seen. In addition to hyperemia and mild hyperplasia of the red pulp in the spleen, mild diffuse mixed inflammatory cell infiltration was detected, and scattered basophilic particles were also found in this organ. Furthermore, mild-to-moderate multiple focal mixed inflammatory cell infiltration were observed in the interstitium of the pancreas, but no evidence for the presence of viral particles was obtained.

**Figure 2:**
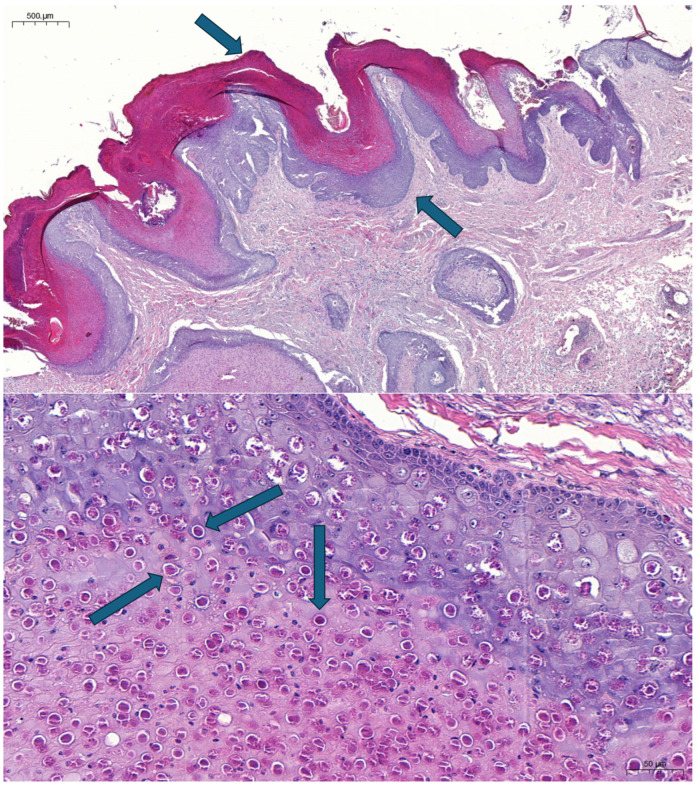
Severe hyperkeratosis (arrow in top position, pointing down) and hyperplasia (arrow in lower position, pointing up) in the epidermis (bar, 500 μm) (**top panel**). Eosinophilic intracytoplasmic inclusions (arrows point to examples) in the epithelial cells; hematoxylin and eosin staining (bar, 50 μm) (**bottom panel**).

In the small intestine, the superficial layers of mucosa were autolyzed and necrotized. Diffuse mixed inflammatory cell infiltration was visible in the lamina propria, and several foci reminiscent of virus particle aggregations were observed. Multiple granulomatous mixed inflammatory cell infiltrations were also seen in the deeper layers of the intestinal wall (including the muscle layers and the serous membrane). Multiple mixed interstitial inflammatory cell infiltration was seen in the lung section, mainly in the areas below the pleura, and some basophilic foci could be found in these sections. Peribronchial lymphofollicular infiltration/proliferation was detected in one area. Mild-to-moderate dermal oedema and mixed inflammatory perivascular or interstitial cell infiltration were seen in all skin sections. Large amounts of eosinophilic cytoplasmic inclusion bodies were observed in most cells in the middle and superficial layers of the hyperplastic epidermis (Figure 2).

**Figure 3:**
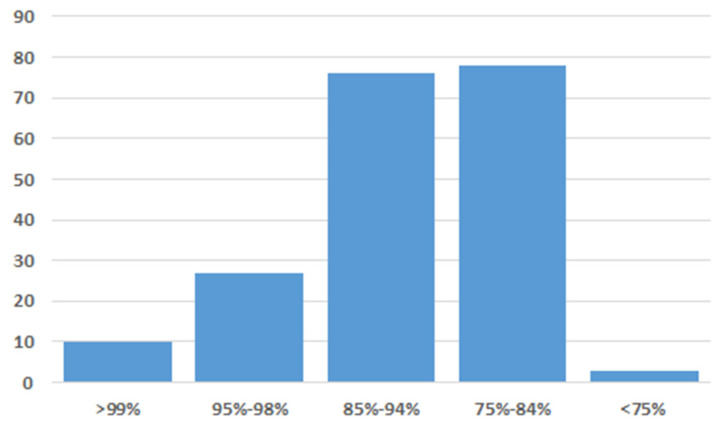
Range and frequency of short DNA polymerase gene sequence identities when comparing the crane poxvirus to other avian poxviruses in GenBank.

Based on the histopathological findings, specific diagnosis was performed to confirm the suspected avipoxvirus infection. Skin and blood samples were used to test for poxvirus DNA, but only the skin tissue sample gave a positive PCR result. PCR product of the expected size was obtained, and its nucleotide sequence was determined via Sanger sequencing. A BLAST engine query of the resulting partial gene sequence in GenBank yielded several hits for DNA polymerase genes of different avipoxviruses (sequence identity range, 74 to 99%; Figure 3).

### 3.2. Genome, Phylogeny, and Classification

**Figure 4:**
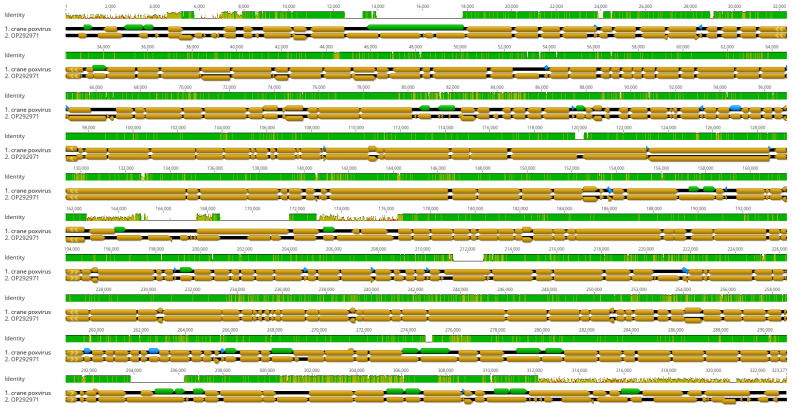
Structural alignment of the annotated genome of crane poxvirus (brown arrows, top) and Cook’s petrel poxvirus (brown arrows, bottom). Blue and green arrows indicate additional ORFs that are missing from the flamingo poxvirus used in the original annotation. Sequence similarity along the two genomes is shown above the alignment (green and olive bar).

The viral genome was determined from specimens collected post-mortem without prior virus isolation and propagation in embryonated egg and cell culture. A large fragment of the viral genome was assembled from a total of 722,646 reads (at a sequencing depth of 229×), which represented ~0.6% of the entire metagenomic assemblage of the skin lesion. The assembled crane avipoxvirus genome was 306,477 bp long and was predicted to encode 313 genes. The ITR motifs remained unresolved. The proportion of forward and reverse orientation genes was similar (forward, n = 181; reverse, n = 132). Nearly a quarter of genes (n = 82, 26.2%) was predicted to encode hypothetical proteins whose function is still unknown. Gene length varied between 93 nt and 5808 nt. Many gene orthologs occurred in multiple copy numbers; the ankyrin repeat family protein coding gene was the most abundant (n = 55); this was followed by the N1R/p28 family protein coding gene (n = 13). Supplementary Table S1 summarizes the main features of the annotated genome, and Figure 4 depicts a simplified version of the annotated viral genomes of crane poxvirus and Cook’s petrel poxvirus, a closely related virus isolate [23].

**Figure 5:**
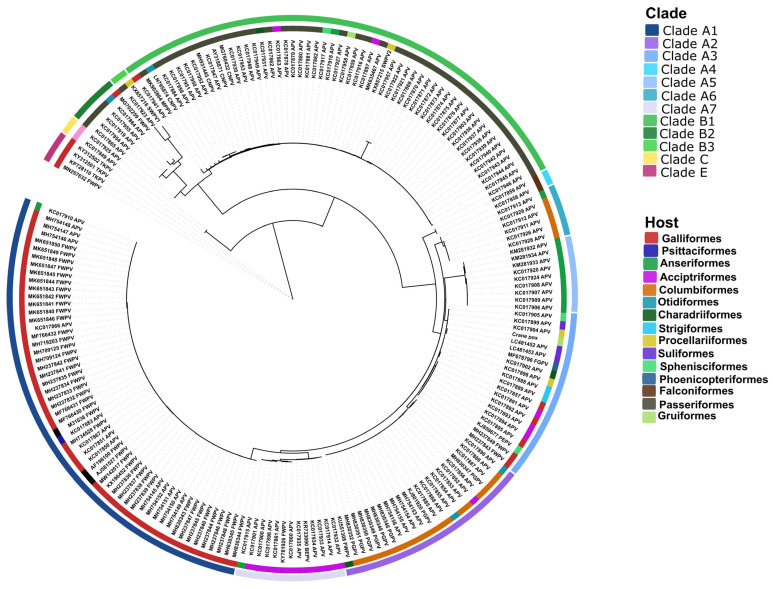
Phylogenetic relationship among avipoxviruses based on the DNA polymerase gene. Color codes indicate the genetic clade (outer circle) and the host origin (inner circle).

Phylogenetic analysis was carried out using the DNA polymerase gene (Figure 5). Nearly 200 sequences were downloaded for phylogenetic analysis. The reference sequences represented the major genetic clades and sub-clades, including subclade A1 to A7, subclade B1 to B3, clade C, and clade E. Amongst the most closely related strains, we found the flamingo origin avipoxvirus (99%), and similar sequence identities were seen with subclade A3 isolates (range, 98.3 to 100%). Of interest, subclade A3 avipoxviruses originated from numerous avian orders, including Galliformes, Strigiformes, Accipitriformes, Procellariiformes, Suliformes, Charadriiformes, Procellariiformes, Sphenisciformes, and Phoenicopteriformes. Additionally, the study virus was detected in a Gruiformes bird. It is notable that the partial gene sequences of another Eurasian crane origin poxvirus strain, K141, isolated in Germany in the mid-2000s, shared over 99% identity to our study virus along a 564 bp fragment of the 4b gene (GenBank#, KF956000) and along a 1888 bp fragment of the fpv140 (GenBank#, KF955996) locus.

## 4. Discussion

The Eurasian crane was a nesting species of the avifauna in Hungary until the early 20th century. Over the following century, breeding cranes disappeared, and the species was considered a migratory bird, which typically visited Hungary as a stopover on the Baltic–Hungarian migratory route. According to the latest reports, between 160,000 and 195,000 cranes could have visited Hungary each year during the autumn migration period [24]. However, in recent years, cranes were observed to breed again in parts of Hungary, albeit sporadically [25]. The population size of migratory birds can be seriously affected during their migration from nesting to wintering sites and vice versa. In a multi-year study reported from Germany, the most common causes of deaths recorded for 161 Eurasian crane carcasses were traumatic injuries (62.9%), organophosphate intoxication (16.8%), and infection (14.4%). Poxvirus infection was identified in a total of three (1.8%) crane carcasses [26]. However, the question on how these infections contribute to crane mortality remains open. It is speculated that the cutaneous form of avipoxvirus infection, especially when lesions are small, is less damaging to birds, and the lesions typically heal within 3–4 weeks of infection [27]. On the contrary, extended, large lesions may severely impair some abilities of affected birds, including vision, breathing, feeding, and water uptake. Thus, even if cutaneous poxvirus infection itself is not fatal, the risk of starvation, dehydration, accidents, and predation may pose a considerable risk to affected birds [3].

In the present study, we report an acute avipoxvirus infection in a Eurasian crane transported to the zoo hospital in Budapest, Hungary. After the rescue, the bird died during the rehabilitation phase. The observed lesions, which accumulated on the featherless parts of the head, were very typical of cutaneous pox. To confirm this, we performed gross pathological examination, histopathology, virus-specific PCR, and viral genome sequencing. Gross pathology showed extensive involvement of internal organs, and histopathology uncovered signs of acute infection with generalized granulomatous inflammation detectable in the kidney, liver, heart muscle, intestinal wall, lung, and spleen. No samples were taken from these organs and tissues; hence, the role of avipoxviruses in the organ-specific lesions remained unconfirmed. Diagnostic PCR targeted the DNA polymerase gene, commonly used for both confirmation of infection and epidemiologic surveillance [5,8]. We sequenced the amplified gene fragment, and a preliminary GenBank query via the Blast engine resulted in numerous avipoxvirus hits.

Next, we sequenced the whole genome to obtain additional information about the viral genome. No virus isolation was carried out prior to sequencing; thus, the data were generated using a classical metagenomics approach from the pathological specimen. This required large amounts of input DNA during sequencing runs. In the end, only 0.6% of the ~127 million sequence reads mapped to the reference avipoxvirus genome. This low yield of avipoxvirus-specific reads from tissue samples is not unexemplified [28,29,30,31]. At this point, it is worth noting that our success was achieved at relatively high costs. The reagent cost of sequencing reached 1200 USD at the time the experiments were performed on a NextSeq500 platform. As of October 2024, when our paper was finalized, this cost could be as low as 140 USD, or even less on advanced, extremely high-throughput sequencing platforms. Thus, as sequencing equipment continues to evolve, and as the relative costs of sequencing runs (i.e., per base expenses) further decreases, the cost–benefit ratio of metagenomics, coupled with whole-genome assembly from poxviral lesions, will likely make it a more feasible option for the disease surveillance of avian pox. The targeted enrichment of avipoxvirus DNA before whole-genome sequencing could serve as an alternative approach to generating high-quality sequence reads at a reduced price.

The obtained near-complete genome sequence permitted the comparison of our virus with an unrelated crane poxvirus, strain K141, whose genome was partially sequenced, although its DNA polymerase gene sequence was not available. This comparison showed a very high genetic similarity with our more recent crane poxvirus; however, the concatenated sequence from this earlier GenBank record represented less than 1% of the complete genome; thus, similarities in gene content remain unknown. Structurally, the flamingo origin avipoxvirus genome and our newly determined crane avipoxvirus genome differed slightly from each other, although excessive changes were seen in some parts of the genome, accompanied by gene gains and losses, together with an increase in sequence divergence. Gene gains and losses are common features of the evolution of poxviral genomes, and these features may be critical for host selection and adaptation [32]. Nonetheless, at present, the mode and frequency of gene gains and losses in avipoxviruses is far from fully understood. The question of whether our crane poxvirus has newly evolved from a flamingo origin avipoxvirus or is a closely related avipoxvirus, emerging via sequential gene gains and losses, cannot be answered at this stage. The bioinformatic pipeline we utilized in this study did not identify the presence of multiple genome variants in the sampled skin tissue. In this respect, the fact that our metagenomic sampling was performed only from skin tissues seems to be a limiting factor. Regardless of these apparent limitations, host adaptation is an intriguing research topic, in terms of genomic content, which deserves further investigation. The availability of the near-complete crane poxvirus genome we determined in this study may go on to contribute to relevant research.

Phylogenetic analysis was performed using the DNA polymerase gene: the Hungarian crane poxvirus genotyped subclade A3. This subclade contains poxviruses originating from at least 10 avian orders, including Galliformes, Strigiformes, Accipitriformes, Procellariiformes, Suliformes, Charadriiformes, Procellariiformes, Sphenisciformes, Phoenicopteriformes, and now Gruiformes, originating from at least five continents. In particular, several penguin species and giant petrel from Argentina, South Africa, and/or the Antarctica; cormorants from the United States and Japan; lesser flamingo from South Africa; Eurasian eagle owls from South Korea; peafowls, partridges, quails, and turkeys from European and African countries; several birds of prey in Europe; and a crane from Hungary are now on the list of avian species harboring subclade A3 avipoxviruses, suggesting a global distribution of this subclade [8,33,34,35]. With respect to host-spectrum and geographic distribution, subclade A3 is among the most diverse groups within clade A avipoxviruses.

## 5. Conclusions

In summary, in the early period of avipoxvirus research, the genetic classification of avipoxviruses was typically preceded by virus isolation and propagation in embryonated egg or cell culture. However, this approach is challenging and time-consuming. Therefore, the direct whole-genome sequencing of avipoxviruses from clinical/pathological samples is an attractive alternative form of virus characterization. The data presented here reinforce the idea that metagenomics, coupled with viral genome assembly directly from tissue samples, without prior enrichment, could be a convenient and feasible alternative form of avipoxvirus surveillance in wild birds and domestic poultry.

## Data Availability

The viral metagenomic sequence reads used to determine the genome of Avipox-virus common crane-1 were deposited under the Bioproject ID PRJNA1074950 (other repository IDs were as follows: Biosample, SAMN39897821; SRA, SRR27940307; Genbank, PP341421).

## Supplementary Materials

The following supporting information can be downloaded at https://www.mdpi.com/article/10.3390/ani15010060/s1: Table S1: Annotation of the crane poxvirus genome.
